# Genomic Diversity and Virulence Potential of High-Priority Critically Important Antimicrobial-Resistant *Escherichia coli* from Pork and Chicken Retail Meat

**DOI:** 10.3390/pathogens15040438

**Published:** 2026-04-18

**Authors:** Hernán D. Nievas, Camila Aurnague, Elisa Helman, Raúl E. Iza, Magdalena Costa, Oliver Mounsey, Matthew B. Avison, Lucía Galli, Fabiana A. Moredo

**Affiliations:** 1 Laboratorio de Bacteriología y Antimicrobianos, Facultad de Ciencias Veterinarias, Universidad Nacional de La Plata, La Plata 1900, Buenos Aires, Argentina; hnievas@fcv.unlp.edu.ar (H.D.N.); caurnague@fcv.unlp.edu.ar (C.A.); riza@fcv.unlp.edu.ar (R.E.I.); fmoredo@fcv.unlp.edu.ar (F.A.M.); 2 Laboratorio de Inmunoparasitología, Facultad de Ciencias Veterinarias, Universidad Nacional de La Plata, La Plata 1900, Buenos Aires, Argentina; ehelman@fcv.unlp.edu.ar; 3 IGEVET—Instituto de Genética Veterinaria “Ing. Fernando N. Dulout” (UNLP-CONICET La Plata), Facultad de Ciencias Veterinarias, Universidad Nacional de La Plata, La Plata 1900, Buenos Aires, Argentina; mcosta@fcv.unlp.edu.ar; 4 School of Biochemistry and Biomedical Sciences, University of Bristol, Bristol BS8 1TD, UK; om9055@bristol.ac.uk (O.M.); matthewb.avison@bristol.ac.uk (M.B.A.)

**Keywords:** ExPEC, ESBL, CTX-M-2, CTX-M-55, ST101, ST117, virulence factor genes, pork and chicken meat

## Abstract

The occurrence of *Escherichia coli* resistant to high-priority critically important antimicrobials (HPCIA) in the food chain is a growing concern for food safety and public health. This study aimed to evaluate whether HPCIA-resistant *E. coli* isolated from pork and chicken meat at retail markets in La Plata, Buenos Aires, Argentina, exhibit source-associated genomic differentiation through whole-genome sequencing. The isolates displayed a polyclonal population structure, encompassing multiple phylogenetic groups and sequence types. Virulence gene profiles were highly diverse, with chicken-derived isolates harbouring a substantially higher number of virulence genes than pork isolates. Notably, one pork isolate carried a complete set of virulence genes characteristic of diarrheagenic *E. coli*. Single Nucleotide Polymorphism-based phylogenetic analysis revealed several closely related subclusters, including strains recovered from both pork and chicken meat from the same retail markets, suggesting recent clonal sharing or cross-contamination at the point of sale. These findings highlight the circulation of genetically diverse HPCIA-resistant *E. coli* in retail meat, underscoring the potential public health risk and the importance of monitoring resistance and virulence determinants throughout the food production chain.

## 1. Introduction

Antimicrobial resistance (AMR) has emerged as a major public health concern in the 21st century [1,2]. The excessive use of antimicrobials as growth promoters in farm animals and their misuse in human and veterinary medicine have led to the spread of resistant bacteria and the ineffectiveness of these drugs [3,4]. To address this issue, under the One Health concept, the World Health Organization, in collaboration with other relevant agencies, has decided to reclassify antimicrobials used in both human and veterinary medicine according to their importance. Within the scale, the high-priority critically important antimicrobials (HPCIAs) for human and veterinary medicine include third and fourth-generation cephalosporins, quinolones, phosphonic acid derivatives, and polymyxins [5].

HPCIAs are essential for the treatment of severe *Escherichia coli* infections in humans. Third-generation cephalosporins (3GCs) are widely used to treat urinary tract, bloodstream, and intra-abdominal infections [6], while quinolones are among the most frequently prescribed antimicrobial classes in human medicine and are also extensively used in veterinary practice [7,8]. In addition, fosfomycin has gained relevance as a last-resort therapeutic option against multidrug-resistant (MDR—defined as acquired resistance to at least one agent in three or more antimicrobial classes) *E. coli* infections [8,9]. Resistance to these antimicrobial classes is often associated with multidrug resistance phenotypes, significantly reducing therapeutic options. Therefore, monitoring resistance to these HPCIA classes in foodborne *E. coli* is crucial for assessing potential public health risks and understanding the dissemination of clinically relevant resistance determinants along the food chain.

*Escherichia coli* is a ubiquitous bacterium inhabiting a wide range of hosts, including humans, birds, and mammals, and can reach humans through multiple transmission routes, particularly via the food chain. It is also commonly detected in environmental reservoirs such as water bodies and sediments, as well as on food-contact utensils and surfaces [10,11]. Due to its widespread distribution, frequent exposure to antimicrobial selective pressure, and high genomic plasticity, *E. coli* is widely used as an indicator organism in AMR surveillance studies [12,13].

Based on genetic markers, *E. coli* can be divided into eight main phylogenetic groups: A, B1, B2, C, D, E, F and clade I [14,15]. Also, derived from multilocus sequence typing, numerous sequence types (STs) and clonal complexes have been described. Among them, high-risk clones (HiRCs) are defined as globally disseminated lineages that harbour multiple AMR determinants, exhibit enhanced fitness and pathogenicity, efficiently transmit between hosts, persist for prolonged periods (often exceeding six months), and can cause severe and/or recurrent infections [14]. To date, the most frequently reported HiRCs are ST131, 1193 and 38. Further international disseminated lineages include ST69, ST10, ST405, ST95, ST648, and ST73, which have been detected in both hospital- and community-associated infections [16].

*E. coli* strains are classified into commensal strains, intestinal pathogenic *E. coli* (IPEC), and extraintestinal pathogenic *E. coli* (ExPEC) [17]. Commensal strains form part of the normal intestinal microbiota and contribute to host homeostasis [18]. IPEC comprise enteropathogenic *E. coli* (EPEC), enterotoxigenic *E. coli*, enteroinvasive *E. coli*, enteroaggregative *E. coli*, diffusely adherent *E. coli*, and Shiga-toxin producing *E. coli* (STEC), all of which are associated with diarrhoeal disease in humans [19]. Among these, enterohemorragic *E. coli*—a subgroup of STEC—represents a major public health concern due to the severity of the symptoms caused, particularly in infants and young children [20]. ExPEC strains, which also originate from the intestinal microbiota, can cause a wide range of extraintestinal infections and are grouped according to the clinical syndromes they cause, such as avian pathogenic *E. coli* (APEC), uropathogenic *E. coli*, neonatal meningitis *E. coli*, and septicemia-associated *E. coli* [21].

In contrast to the more clearly defined virulence factors exhibited by IPEC strains, ExPEC strains exhibit greater genotypic heterogeneity, facilitating the sharing of virulence factors among diverse pathotypes [22]. Numerous studies focus on identifying different ExPEC-associated virulence factors, such as those related to adhesion (*fim*, *afa*, *dra*, *pap*, *sfa*, *foc*, *iha*, *mat*, *csg*, *fdeC*, *lpfA*), iron uptake (*iucC*, *irp2*, *iroN*, *chu*, *hma*, *sitA*, *ireA*, *fyuA*), protectins (*traT*, *kps*, *neu*, *iss*, *cvaC*, *ompT*), or invasins (*shiA*, *shiB*, *tia*, *ibe*), and toxins (*hlyA, hlyE*, *hlyF*, *astA*, *pic*, *sat*, *vat*) [12,21,23,24,25,26]. One of the elements associated with *E. coli* virulence is the ColV plasmid [27]. These plasmids typically encode multiple genetic loci, including iron acquisition and transport systems, outer membrane vesicles, and proteases, as well as serum-resistance genes, thereby conferring a broader repertoire for survival and increased virulence [28].

One route of *E. coli* transmission to humans is through meat. Globally, poultry and pork represent the most widely produced and consumed meat types. In 2021, chicken and pork each accounted for approximately 34% of global meat production, with poultry showing the highest growth rates over the past two decades [29]. Consumption of both meat types is expected to continue increasing worldwide [30]. In Argentina, beef remains the most widely consumed meat, followed by poultry and pork, underscoring the importance of food animal production systems as potential reservoirs of antimicrobial-resistant bacteria [31].

The objective of this study was to evaluate the hypothesis that HPCIA-resistant, potentially pathogenic *E. coli* isolates recovered from pork and chicken meat exhibit source-associated genomic differentiation, indicative of reservoir-specific distribution patterns, using whole-genome sequencing (WGS).

## 2. Materials and Methods

### 2.1. HPCIA Resistant E. coli

A total of 230 fresh meat samples were collected from 54 retail markets located in La Plata, Buenos Aires, Argentina, between February 2022 and April 2024. Retail markets were randomly selected across the city. Sampling was performed throughout the study period without restriction to specific months or seasons in order to capture routine retail conditions. Of the total samples, 138 corresponded to pork and 92 to chicken meat. From these samples, 172 HPCIA-resistant *E. coli* isolates were recovered and included in this study, comprising 85 isolates from pork (EC-P) and 87 from chicken (EC-C). Isolation was performed using a selective approach targeting resistance to third-generation cephalosporins, ciprofloxacin, or fosfomycin. Briefly, 25 g of each sample was homogenized with 225 mL of buffered peptone water (Britania, Buenos Aires, Argentina) and incubated overnight at 37 °C. Subsequently, 30 μL of the enrichment culture was inoculated onto MacConkey agar supplemented with 4 mg/L cefotaxime or 1 mg/mL ciprofloxacin, and onto eosin methylene blue agar with a 200 μg fosfomycin disc, followed by incubation at 37 °C for 18 h. From each selective plate, one colony with morphology compatible with *E. coli* was selected; when clearly distinct morphotypes were observed, up to two colonies were recovered to minimize selection bias. Isolates were subcultured on tryptone soy agar (Britania) and identified by conventional biochemical tests. Confirmed *E. coli* isolates were preserved in the LaByAn (Laboratorio de Bacteriología y Antimicrobianos) strain collection at −20 °C in brain–heart infusion broth (Britania) supplemented with 30% glycerol (Anedra, Buenos Aires, Argentina).

The resistance profiles of the EC-P and EC-C to HPCIAs were determined by the disk diffusion method according to Clinical Laboratory Standards Institute recommendations [32]. The HPCIAs tested were cefotaxime 30 μg, ceftazidime 30 μg, ciprofloxacin 5 μg and fosfomycin 200 μg. Commercial antibiotic disks were acquired from Britania. Clinical breakpoints and interpretative category definitions (susceptible and not susceptible—resistant and intermediate) were those recommended for human isolates [32].

### 2.2. WGS and Bioinformatics

WGS of the 172 HPCIA-resistant *E. coli* isolates was performed on an Illumina NovaSeq 6000 (Illumina, San Diego, CA, USA) using a 250-base-pair paired-end protocol as Mounsey et al. 2024 [33]. The sequences were analysed on the Galaxy platform version 2.4.0, ResFinder version 4.7.2, Mobile genetic element version V1.0.3, EzClermont version 0.4.0, MLST version 2.0, iTree V3 and iTOL v7 for different analyses [34,35,36,37,38,39,40].

### 2.3. Core Genome Single Nucleotide Polymorphism (SNP) Phylogeny

Phylogenetic analysis was performed using the Galaxy platform. A core genome alignment was generated using Roary (Galaxy Version 3.13.0+galaxy3) with a BLASTp identity threshold of 95%. SNPs were extracted using snp-sites (Galaxy Version 2.5.1+galaxy0). A maximum likelihood phylogenetic tree was inferred using IQ-TREE (Galaxy Version 2.4.0+galaxy1) under the best-fit substitution model selected by ModelFinder according to the Bayesian Information Criterion. Branch support was assessed using 1000 replicates of the ultrafast bootstrap approximation. Pairwise SNP distances between isolates were calculated using snp-dists (Galaxy Version 0.8.2+galaxy0). The resulting tree was midpoint-rooted for visualisation purposes and annotated using iTOL (https://itol.embl.de/) [41].

### 2.4. Statistical Analysis

Associations between the isolate group (EC-P vs. EC-C) and the presence of virulence factors, AMR genes, multidrug resistance profiles and ST were evaluated using generalised linear models with a binomial distribution and a logit link function. The presence/absence of each trait was specified as the response variable and the isolate group as the explanatory variable. Statistical significance was assessed using Wald tests. The magnitude of the association was expressed as the Odds Ratio (OR) with a 95% confidence interval.

To assess the overall dissimilarity in virulence gene content between isolates from different sources, gene presence/absence data were converted into a binary matrix. A Jaccard distance matrix was calculated to estimate compositional dissimilarity (vegdist function). Differences in virulence profiles between groups were evaluated using Permutational Multivariate Analysis of Variance (PERMANOVA) (adonis2 function) with 999 permutations. Pairwise post hoc comparisons were conducted using a custom PERMANOVA function with Holm’s correction for multiple testing.

Multivariate dispersion and clustering of isolates based on virulence gene content were visualised using Principal Coordinates Analysis (PCoA). Additionally, the prevalence of virulence genes and their association with phylogenetic groups were visualised using heatmaps generated with the ggplot2 package.

All statistical analyses were performed using the R software environment (version 4.3.1) [42]. The threshold for statistical significance was established at *p* < 0.05 for all tests.

## 3. Results

The HPCIA-resistant *E. coli* genomes were clustered into two groups based on their sources: pork and chicken meat. The distribution of AMR gene variants according to isolate origin is shown in Table 1.

### 3.1. Molecular Characterisation of the AMR Profile

#### 3.1.1. Cefotaxime Resistance (CTX-R): Extended-Spectrum β-Lactamase (ESBL) and Plasmid-Mediated AmpC (p-AmpC)

Among the 108 cefotaxime-resistant *E. coli* isolates (35 EC-P and 73 EC-C), resistance mediated by the *bla*_CTX-M_ genes was predominant. In addition, 2.3% of the isolates carried the *bla*_CMY-2_ gene. The most frequently detected variants were *bla*_CTX-M-2_ (28.54%), followed by *bla*_CTX-M-55_ (13.4%), *bla*_CTX-M-15_ (7.5%), *bla*_CTX-M-14_ (7%), *bla*_CTX-M-8_ (2.3%), *bla*_CTX-M-65_ (1.2%), and *bla*_CTX-M-27_ (0.6%). *bla*_CTX-M-2_ and *bla*_CTX-M-55_ were the most prevalent variants in EC-C and EC-P, respectively.

We observed a very strong association between *bla*_CTX-M-2_ and chicken meat (*p* < 0.05; OR = 0.06), suggesting that it is 16 times more likely to be detected in chicken meat than in pork.

#### 3.1.2. Ciprofloxacin Resistance (CIP-R): Plasmid-Mediated Quinolone Resistance (PMQR) and Mutations Altering the Quinolone Resistance-Determining Regions (QRDR)

Of the total 85 EC-P isolates, 71 were ciprofloxacin not-susceptible (62 resistant and nine intermediate). Similarly, among the 87 EC-C isolates, 73 were ciprofloxacin not-susceptible (66 resistant and seven intermediate). Among the 144 ciprofloxacin not-susceptible *E. coli* isolates, QRDR mutations, which were detected in 40 EC-P and 47 EC-C isolates, were the primary cause.

A significant association was found between the source of HPCIA-resistant *E. coli* and the prevalence of two QRDR mutation/PMQR gene combinations (Table 1). The combination *qnrS1*/*gyrA* was exclusively associated with EC-P (OR > 5). The combination *qnrB19*/*gyrA*/*parC* was more common in EC-C than EC-P (OR = 0.19).

#### 3.1.3. Fosfomycin Resistance (FOS-R): Mobile Genes *fosA3*, *fosA7*, and *fosL1*

Among the 57 fosfomycin-resistant *E. coli* isolates (20 EC-P and 37 EC-C), FOS-R was mediated by the mobile resistance genes *fosA3*, *fosA7*, and *fosL1*. No statistically significant differences were observed in the distribution of these genes between pork and chicken isolates (*p* > 0.05).

#### 3.1.4. Frequent Associations of HPCIA-Resistance Genes

Of the 172 HPCIA-resistant *E. coli* isolates, 40 (23.25%) were MDR carrying resistance genes to cefotaxime, ciprofloxacin, and fosfomycin. Of these, eight isolates (8/172, 4.65%) were EC-P and 32 (32/172, 18.6%) were EC-C (Supplementary Materials). This triple resistance profile was significantly more frequent among EC-C than EC-P (*p* < 0.05; OR = 0.18).

In EC-P, the most frequent resistance gene combination was *bla*_CTX-M-55_/*qnrB19/fosA3* (*n* = 4). In contrast, a greater diversity of resistance-gene combinations was observed among chicken meat isolates. The most frequent profiles included *bla*_CTX-M-2_/*gyrA/parC/fosL1* (*n* = 7).

Across all isolates, the most common co-occurrence involved 3GC resistance (3GC-R) alongside CIP-R, followed by the combination of CIP-R and FOS-R. The 3GC-R/CIP-R combination was significantly associated with EC-C (*p* < 0.05). In contrast, in the CIP-R/FOS-R combination, no significant association with the meat source was found (*p* > 0.05). No isolates carrying the 3GC-R/FOS-R combination were detected.

### 3.2. Phylogenetic Analysis

#### 3.2.1. Phylogenetic Groups

**Figure 1:**
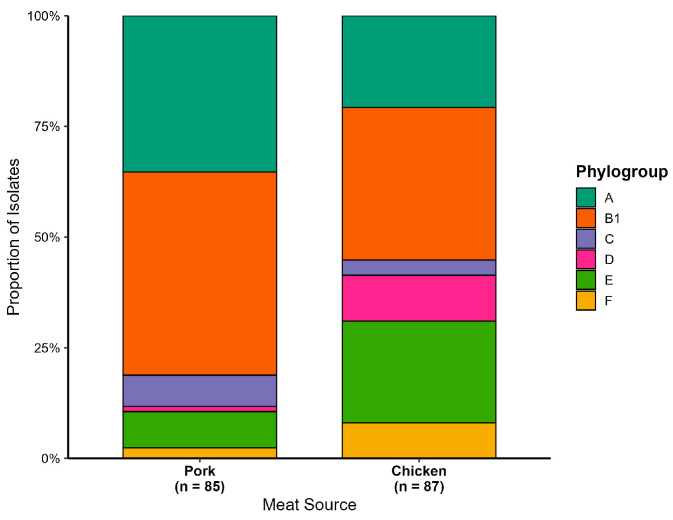
Distribution of phylogenetic groups of HPCIA-resistant *E. coli* isolated from pork and chicken meat. The stacked bar chart illustrates the proportion of isolates assigned to each Clermont phylogroup (indicated by colour) within each meat source. The total number of isolates analysed for each meat source is shown in parentheses on the *x*-axis.

The 172 HPCIA-resistant *E. coli* isolates were grouped into six phylogenetic groups: B1 (*n* = 68), A (*n* = 49), E (*n* = 27), D (*n* = 27), C (*n* = 9), and F (*n* = 9) (Figure 1). Notably, no isolates belonged to the B2 group.

A significant source-dependent distribution of phylogenetic groups was observed. Phylogroup A was significantly associated with EC-P (*p* < 0.05; OR = 3.0). Conversely, phylogroup D, commonly associated with greater pathogenic potential, was significantly associated with EC-C (*p* < 0.05; OR = 0.1).

#### 3.2.2. Sequence Type

A total of 62 STs were identified among the 172 HPCIA-resistant *E. coli* isolates, including 44 STs in EC-P and 37 in EC-C. Nineteen STs were shared between both sources: ST10, ST48, ST57, ST58, ST88, ST93, ST101, ST117, ST155, ST162, ST196, ST206, ST224, ST345, ST602, ST744, ST1196, ST1431, and ST1582.

A total of 42 (24.4%) HPCIA-resistant *E. coli* isolates were classified as belonging to recognised emerging ExPEC linages. The most frequently detected STs, in descending order, were ST744 (16.6%), ST101 (14.3%), ST48 (14.3%), ST10 (11.9%), ST117 (11.9%), ST88 (9.5%), and ST58 (7.1%). Among EC-P isolates, the most prevalent were ST101, ST10, and ST48, whereas in EC-C isolates, ST117 and ST744 predominated (Supplementary Materials).

#### 3.2.3. Virulence Factor Genes (VGs)

A total of 65 VGs were identified among the HPCIA-resistant *E. coli* isolates analysed. Table 2 summarises the VGs shared between HPCIA-resistant *E. coli* isolated from pork and chicken meat.

Several VGs were detected exclusively in EC-P (Supplementary Materials). These included adhesin-associated genes such as *sfaD* (*n* = 3), *sfaE* (*n* = 2), *focC* (*n* = 4), *focG* (*n* = 4), *focL* (*n* = 1), *aaiC* (*n* = 1), *mrka* (*n* = 12), as well as protectin-related genes including *clpk2* (*n* = 6), *katp* (*n* = 5), *capU* (*n* = 3), *espY* (*n* = 6), *mcbA* (*n* = 1), and *mcmA* (*n* = 6). In contrast, the adhesin genes *afa* (*n* = 4) and *neuC* (*n* = 4) were detected exclusively in EC-C.

Notably, one EC-P isolate harboured multiple VGs characteristic of diarrheagenic strains, including *nleA*, *nleB*, *tir*, *espA*, *espB*, *espJ*, *eae-g01-gamma_1*, *cif*, *tccp* and *ehxA.* Based on this VG profile, the isolate was classified as EPEC.

**Figure 2:**
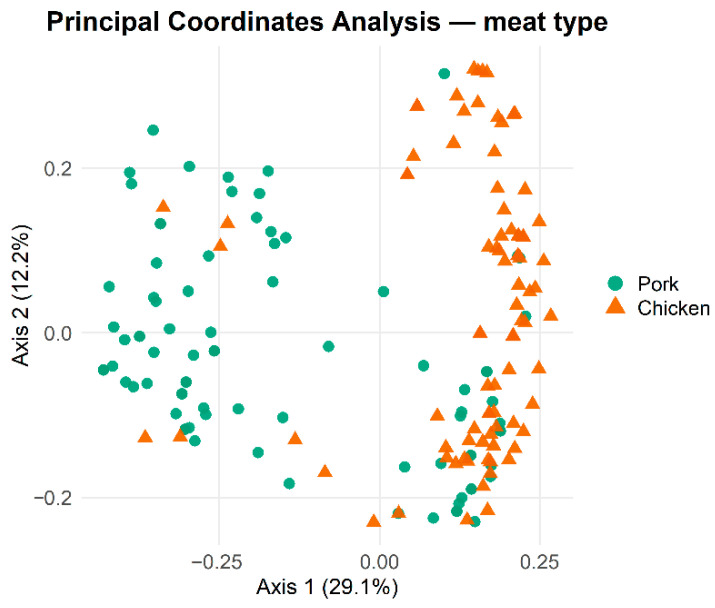
PCoA. Each point represents an individual HPCIA-resistant *E. coli*, with colours and shapes indicating the source (pork or chicken meat). The first two PCoA axes explain 29.1% (PCoA1) and 12.2% (PCoA2) of the total variation, respectively. Differences between groups were statistically supported by PERMANOVA (Jaccard distance; 999 permutations).

Figure 2 illustrates the clustering of isolates by the presence or absence of VGs, with a clear separation by source of isolation. EC-P exhibited greater heterogeneity compared to EC-C.

**Figure 3:**
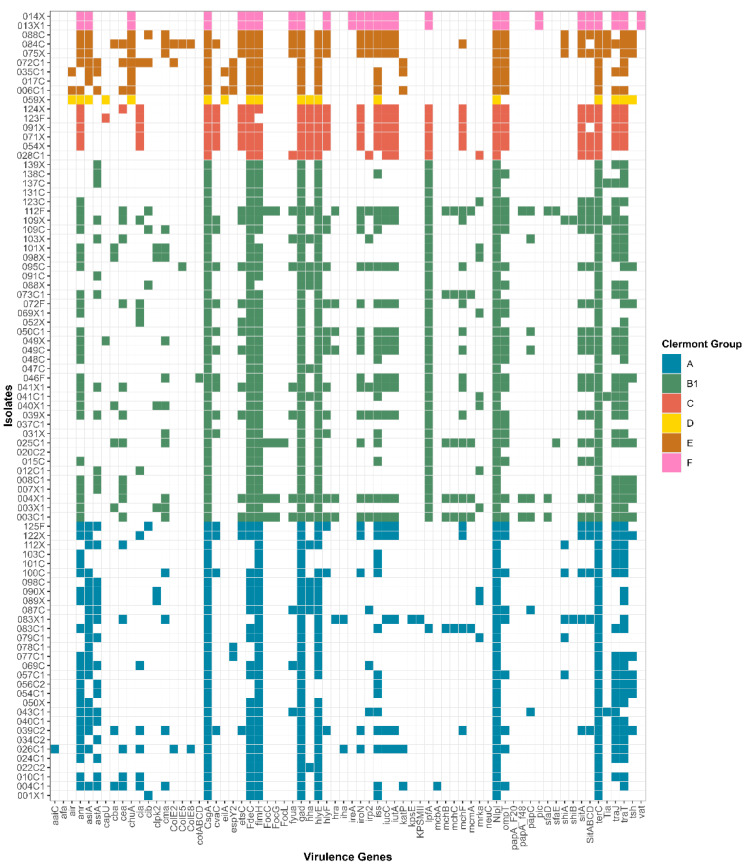
Distribution of VGs and phylogenetic classification of HPCIA-resistant *E. coli* isolates from pork meat. The heatmaps display the presence (coloured cells) or absence (white cells) of virulence genes (*x*-axis) across individual isolates (*y*-axis). The colour coding corresponds to the Clermont phylogroup assignment: A (blue), B1 (green), C (red), D (yellow), E (brown), and F (pink).

**Figure 4:**
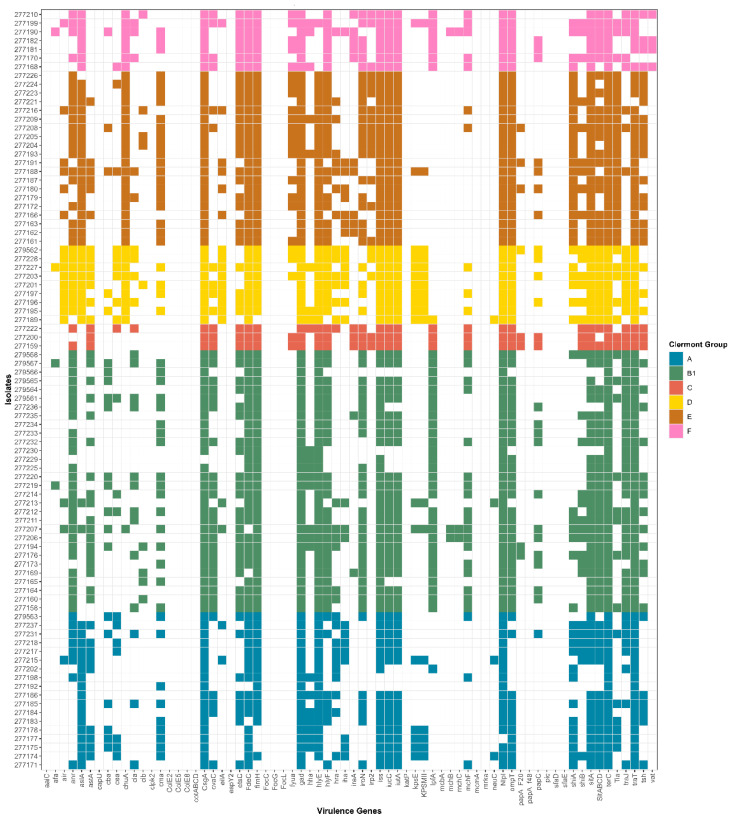
Distribution of VGs and phylogenetic classification of HPCIA-resistant *E. coli* isolates from chicken meat. The heatmaps display the presence (coloured cells) or absence (white cells) of virulence genes (*x*-axis) across individual isolates (*y*-axis). The colour coding corresponds to the Clermont phylogroup assignment: A (blue), B1 (green), C (red), D (yellow), E (brown), and F (pink).

Figure 3 and Figure 4 present the distributions of VGs and phylogenetic groups by isolate source. Overall, EC-P harboured fewer VGs than EC-C.

### 3.3. Core Genome SNP Analysis

**Figure 5:**
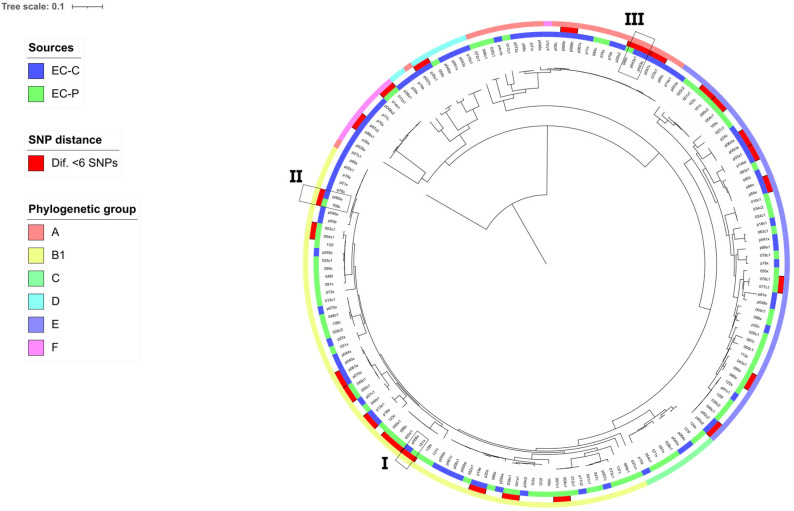
HPCIA-resistant *E. coli* isolated from pork and chicken meat phylogenomic SNP tree with circular heatmap shows (outside-in reading rings): sources of *E. coli* (first inner ring); SNP distance (second inner ring); and phylogenetic group (third inner circle). Numbers I, II and III designate the three clusters that include strains of both sources.

The SNP maximum likelihood tree of HPCIA-resistant *E. coli* isolated from pork and chicken meat is presented in Figure 5. Six clusters related to the phylogenetic group and 24 clones with differences ≤ 6 SNPs were observed. Numbers I, II and III denote the clones including both EC-P and EC-C isolates. In the three cases, both were isolated from the same retail market (Supplementary Materials).

Subcluster I: one EC-P and one EC-C serogroup O83:H7, ST196, harbour *bla*_CTX-M-15_ and *qnrS1* and 12 VGs (*hlyE*, *cba*, *cma*, *traJ*, *gad*, *traT*, *csgA*, *terC*, *nlpl*, *lpfA*, *fimH*, *fdeC*). The isolates differed by six SNPs.

Subcluster II: one EC-P and one EC-C, serogroup O non-determining:H21, ST602, harbour *bla*_CTX-M-2_ and *fosA7* with mutations in *gyrA* and *parC* and 25 VGs (*iroN*, *iutA*, *ompT*, *hlyF*, *iss*, *cvaC*, *hlyE*, *astA*, *cia*, *cea*, *traJ*, *iucC*, *sitA*, *gad*, *traT*, *etsC*, *tia*, *csgA*, *terC*, *nlpl*, *lpfA*, *fimH*, *fdeC*, *shiA*, *shiB*). The isolates differed by zero SNPs.

Subcluster III: one EC-P and two EC-C, serogroup O non-determining:H25, ST57, harbour *bla*_CTX-M-2_, *fosL1* and *qnrB19* with mutations in *gyrA* and *parC* and 23 VGs (*iroN*, *fyuA*, *iutA*, *ompT*, *hlyF*, *iss*, *hlyE*, *cib*, *cma*, *iucC*, *sitA*, *gad*, *traT*, *chuA*, *etsC*, *tia*, *csgA*, *terC*, *nlpl*, *fimH*, *fdeC*, *shiA*, *tsh*). The clones differed by two SNPs.

## 4. Discussion

To our knowledge, this is the first study to comprehensively evaluate both AMR patterns and VG profiles of HPCIA-resistant *E. coli* isolated from pork and chicken meat in La Plata, Buenos Aires, Argentina. Considering that 3GC, ciprofloxacin and fosfomycin are classified as HPCIAs for human medicine, the detection of resistance to these agents in foodborne *E. coli* highlights the role of retail meat within the food chain as a potential interface for the circulation of resistance determinants of clinical importance.

In *E. coli*, resistance to 3GC is primarily mediated by ESBL and, to a lesser extent, plasmid-mediated AmpC enzymes. Among ESBL, CTX-M enzymes constitute the most widely disseminated family worldwide, with CTX-M-2, CTX-M-9, CTX-M-14, and CTX-M-15 being among the most frequently reported variants within *Enterobacterales*. In Argentina, clinical studies have shown predominance of the CTX-M-1/15 group, followed by CTX-M-9/14 and CTX-M-2 [43]. Consistent with this regional epidemiological scenario, *bla*_CTX-M_ genes were the main mechanism of 3GC resistance in both EC-P and EC-C isolates analysed in this study, although their distribution differed by source. This source-specific distribution of CTX-M variants could suggest that distinct ecological and epidemiological dynamics may operate within each production chain. In chicken isolates, *bla*_CTX-M-2_ was the most frequently detected variant, in agreement with previous reports from Argentina [44,45], whereas studies from Brazil have described *bla*_CTX-M-55_ as predominant in poultry meat [46]. In pork isolates included in this study, *bla*_CTX-M-55_, *bla*_CTX-M-14_, and *bla*_CTX-M-15_ were the most common variants. Notably, investigations conducted in pig farms in Argentina have identified *bla*_CTX-M-8_ as the dominant ESBL variant [20,33,47], in contrast to the retail pork isolates evaluated in this study. These differences between primary production and retail meat may reflect mixing of animals from multiple sources during slaughter and processing, differential survival or fitness of specific plasmid–host combinations, horizontal transfer of resistance plasmids in processing environments, or introduction of additional lineages during meat distribution and commercialisation.

Quinolone resistance arises through chromosomal mutations and the acquisition of plasmid-mediated determinants [48]. In this study, QRDR mutations were the primary mechanism of ciprofloxacin resistance in both EC-P and EC-C isolates, consistent with reports from Europe and Africa [49,50,51]. Nevertheless, the detection of PMQR alone or in combination with QRDR mutations is epidemiologically relevant. Although PMQR determinants generally confer reduced susceptibility, they may facilitate bacterial survival under subinhibitory exposure and promote the selection of additional chromosomal mutations. Their plasmid-borne nature also enables horizontal dissemination across strains and ecological niches. Notably, specific resistance profiles were associated with source: *qnrS1*/*gyrA* was detected exclusively in EC-P, whereas *qnrB19*/*gyrA*/*parC* predominated in EC-C. These source-associated combinations could suggest differences in antimicrobial selection pressure, plasmid circulation, or clonal backgrounds across production systems. The identification of CIP-R isolates harbouring only PMQR determinants in the absence of detectable QRDR mutations suggests that additional mechanisms or regulatory factors may influence the final resistance phenotype.

Although fosfomycin use is restricted in several European countries and China, it remains widely applied in food animal production in Central and South America, particularly in pig and poultry systems [9], which may contribute to regional differences in resistance patterns. In the present study, fosfomycin resistance was mainly associated with *fosL1* in both pork and chicken isolates, in contrast to reports from Brazil, where *fosA3* predominates in food-derived *E. coli* [23,46,52]. While *fosA3* and *fosA7* were also detected here, their frequencies did not differ significantly between sources, suggesting a broad dissemination of fosfomycin resistance determinants across livestock systems, irrespective of host species. The high overall prevalence observed is worrisome given the increasing clinical relevance of fosfomycin in the treatment of severe infections.

Several studies have reported frequent resistance to 3GC, quinolones, and fosfomycin—alone or combined—among foodborne *E. coli* isolates [23,46,52]. In the present study, the HPCIA-resistant *E. coli* isolates analysed exhibited multidrug resistance and various combinations of clinically relevant resistance genes. However, consistent with the findings of Kocsis et al. [14], none of the isolates carried carbapenemase-encoding genes or exhibited colistin resistance. The coexistence of resistance mechanisms to multiple HPCIAs remains a significant public health concern, as it considerably restricts therapeutic options and promotes the persistence and spread of MDR strains throughout the food chain.

In the present study, most HPCIA-resistant *E. coli* isolates from both pork and chicken meat belonged to phylogroups B1 and A. This distribution is consistent with previous studies on *E. coli* isolated from animal-origin food products, regardless of meat type [53,54]. In contrast, one of the phylogroups more commonly associated with ExPEC, group D [53], was less frequent overall but significantly associated with chicken-derived isolates. This finding suggests that, in the samples studied, chicken meat may serve as a more important reservoir of *E. coli* strains with greater pathogenic potential.

Although phylogroups B1 and A are traditionally considered commensal, they frequently encompass STs of recognised epidemiological relevance. In the present study, ST distribution differed by source: ST101 and ST10 were two of the most prevalent isolates from pork, whereas ST117 and ST744 were more frequent in chicken, although some lineages were shared. The detection of internationally recognised clones highlights the potential dissemination of resistant and pathogenic *E. coli* lineages through the food chain. ST101, despite not being classified as a pandemic clone, has attracted attention due to the emergence of pan-drug-resistant strains and its global distribution [55], with documented associations with extraintestinal infections and the spread of AMR [56]. Similarly, ST10 has been reported in human infections, animal outbreaks, and meat products, and is increasingly regarded as an emerging lineage involved in the dissemination of fluoroquinolone and cephalosporin resistance [46]. ST117, frequently associated with poultry, is commonly MDR, and genotypic similarities between APEC and ExPEC strains, with most human isolates predicted to originate from poultry [57], reinforce the zoonotic potential of this lineage. Finally, ST744 has been recognised as an MDR lineage associated with plasmid-mediated colistin resistance in other settings [58]; although no phenotypic resistance to colistin was observed in our isolates, ST744 presence in retail meat remains epidemiologically relevant due to its capacity to acquire and disseminate clinically important resistance determinants. Collectively, these findings underscore the ecological adaptability and global dissemination of these lineages and support their relevance within a One Health framework.

In addition to clonal background, the virulence potential of these isolates was further explored through VG profiling. The identification of 65 VGs among HPCIA-resistant *E. coli* isolates demonstrates the coexistence of resistance and virulence determinants within food-associated strains. In the present study, VG profiles were highly diverse, with 35.2% of EC-P and 91.9% of EC-C harbouring more than 20 VGs, indicating a markedly higher VG burden in chicken-derived strains. This higher virulence gene content may be explained by the well-recognized role of poultry as a reservoir of avian pathogenic *E. coli* (APEC), which typically harbour a broader repertoire of virulence-associated genes. In addition, many of these determinants are carried by ColV-type plasmids frequently reported in avian isolates, which may facilitate their maintenance and co-selection under antimicrobial pressure. Several VGs exhibited source-specific prevalence, with adhesin- and protectin-associated genes predominating in EC-P, whereas *afa* and *neuC* were more frequent in EC-C. The differential distribution of VGs between pork and chicken isolates further suggests the circulation of source-adapted lineages shaped by distinct ecological and production-related pressures. Functionally, many of the adhesin-, iron acquisition-, and protectin-associated genes identified are known to enhance intestinal colonization, biofilm formation, serum resistance, and persistence in extraintestinal environments. These traits may favour the survival and maintenance of these strains along the production chain, from the animal host to the retail environment. The co-occurrence of these functional traits with multidrug resistance may confer a selective advantage under antimicrobial pressure, facilitating the persistence and dissemination of these lineages in food production systems [26]. Although classical ExPEC-associated VGs are frequently overrepresented among infection-causing lineages, clear genetic boundaries between commensal *E. coli* and facultative ExPEC pathogens remain poorly defined [16]. Notably, eight of the ten markers commonly proposed to differentiate APEC from commensal strains [17] were detected, predominantly among EC-C isolates. While the presence of these markers does not allow for definitive classification as APEC, this pattern is consistent with previous reports describing a higher overall prevalence of VGs in chicken-derived *E. coli* compared with pork isolates [23,59]. Furthermore, many of these virulence determinants have been associated with plasmids involved in avian colibacillosis [60], and their co-occurrence with multidrug resistance has been linked to increased bacterial fitness under antimicrobial selective pressure [26].

Particularly noteworthy was the identification of an EC-P isolate harbouring a complete set of VGs characteristic of EPEC, including *eae*, *tir*, *esp* genes and non-LEE-encoded effectors, supporting its classification as an intestinal pathogenic strain. Although detected in a single isolate, its occurrence in retail meat, in combination with resistance to critically important antimicrobials, highlights the food chain as a relevant reservoir of MDR *E. coli* carrying diverse virulence traits [56]. Overall, irrespective of the specific VG combinations identified, the presence of MDR *E. coli* carrying diverse virulence determinants underscores the importance of the entire production continuum in limiting the dissemination of strains, with potential implications for both public health and animal production.

The SNP-based phylogeny revealed a genetically diverse population of HPCIA-resistant *E. coli* circulating in pork and chicken meat. The presence of six major clusters corresponding to phylogenetic groups A, B1, C, D, E and F indicates that HPCIA resistance is distributed across multiple evolutionary backgrounds rather than being restricted to a single epidemic lineage. This pattern suggests that resistance dissemination is primarily driven by horizontal transfer of mobile genetic elements rather than clonal expansion, consistent with previous reports describing polyclonal populations of resistant *E. coli* in food-producing animals and retail meat [61,62]. Although no universal SNP threshold exists to define isolate sharing and such cut-offs are inherently context-dependent [63], the identification of 24 clones differing by fewer than six core genome SNPs suggests recent common ancestry and potential short-term clonal persistence. Most subclusters were source-specific, supporting the existence of partially independent reservoirs associated with different animal hosts, as was previously described for certain livestock-associated *E. coli* lineages [16,63]. However, three subclusters included isolates from both pork and chicken recovered from the same retail market, displaying nearly identical ARGs and VGs. These findings suggest recent clonal sharing, which may reflect cross-contamination within the retail environment or exposure to a common upstream source during slaughter or processing. However, more studies are needed to confirm this.

## 5. Conclusions

This study provides a genomic overview of HPCIA-resistant *Escherichia coli* circulating in retail pork and chicken meat in La Plata, Argentina. Resistance to 3GC, ciprofloxacin and fosfomycin was widespread and primarily associated with plasmid-borne determinants and chromosomal mutations distributed across multiple phylogenetic backgrounds, indicating a polyclonal population structure. Although several lineages were shared between meat types, the distribution of ARGs, STs, and VGs differed between pork and chicken isolates, suggesting partially independent ecological and epidemiological dynamics within each production chain. Notably, the coexistence of AMR and diverse VG repertoires—including the detection of an isolate carrying a complete EPEC virulence profile—highlights the potential public health relevance of these strains. Furthermore, the identification of nearly identical clones from pork and chicken meat recovered from the same retail markets suggests that the final stages of the food production chain may facilitate the convergence and dissemination of resistant lineages. Together, these findings underscore the importance of integrated genomic surveillance of AMR and virulence in foodborne *E. coli* across the entire production and distribution chain.

## Figures and Tables

**Table 1:** Frequency of distribution of HPCIA-resistance genes among *E. coli* isolated from pork or chicken meat.

**CTX-R**		**Sources** ***E. coli*** **Isolates**					
		**Pork** **(*****n*** **= 85)**		**Chicken Meat (*****n*** **= 87)**		***p*** **-Value**	**OR**
**ESBL**	**p-AmpC**	**n**	**%**	**n**	**%**		
*bla*_CTX-M-2_	**-**	5	5.9	44 ^b^	50.6	**<0.05**	**0.06**
*bla*_CTX-M-8_	-	3 ^a^	3.5	1	1.1	>0.05	3.15
*bla*_CTX-M-14_	-	8	9.4	4	4.6	>0.05	2.16
*bla*_CTX-M-15_	-	8	9.4	5	5.7	>0.05	1.70
*bla*_CTX-M27_	-	0	0	1	1.1	>0.05	ND
*bla*_CTX-M-55_	-	9	10.6	14	16.1	>0.05	0.62
*bla*_CTX-M-65_	-	0	0	2	2.3	>0.05	ND
-	CMY-2	2	2.4	2	2.3	>0.05	1.02
**CIP-R**		**Pork**		**Chicken Meat**		***p*** **-Value**	**Odds Ratio**
**PMQR**	**QRDR**	**n**	**%**	**n**	**%**		
*qnrB19*	-	2	2.4	8	9.2	>0.05	0.23
*qnrS1*	-	8	9.4	3	3.4	>0.05	2.91
-	*gyrA*	0	0	4	4.6	>0.05	ND
-	*parC/parE*	1	1.2	0	0	>0.05	ND
-	*gyrA/parC*	38	44.7	43	49.4	>0.05	0.83
-	*gyrA/parC/parE*	1	1.2	0	0	>0.05	ND
*qnrB19*	*gyrA*	0	0	1	1.1	>0.05	ND
*qnrB19*	*parC*	0	0	1	1.1	>0.05	ND
*qnrB19*	*gyrA/parC*	2	2.3	10 ^c^	11.5	**<0.05**	**0.19**
*qnrS1*	*gyrA*	10	11.8	0	0	**<0.05**	**>5**
*qnrS1*	*gyrA/parC*	5	5.9	2	2.3	>0.05	2.66
*qnrS13*	*gyrA*	1	1.2	0	0	>0.05	ND
*qnrS13*	*gyrA/parC*	1	1.2	1	1.1	>0.05	ND
*qnrS13*	*gyrA/parC/parE*	1	1.2	0	0	>0.05	ND
*OqxB*	*gyrA/parC*	1	1.2	0	0	>0.05	ND
**FOS-R**		**Pork**		**Chicken Meat**		***p*** **-Value**	**Odds Ratio**
		**N**	**%**	**n**	**%**		
*fosA3*		6	7.1	7 ^d^	8	>0.05	0.87
*fosA7*		1	1.2	6	6.9	>0.05	0.16
*fosL1*		13	15.3	24 ^e^	27.6	>0.05	0.47

CTX-R: cefotaxime resistance. ESBL: extended-spectrum β-lactamase. p-AmpC: plasmid-mediated AmpC. CIP-R: ciprofloxacin resistance. PMQR: plasmid-mediated quinolone resistance. QRDR: quinolone resistance-determining regions. FOS-R: fosfomycin resistance. ND: not determined. ^a^: one *E. coli* isolate harbours both *bla*_CTX-M-8_ and *bla*_CTX-M-55_; ^b^: one *E. coli* isolate harbours both *bla*_CTX-M-2_ and *bla*_CTX-M-55_ variants. ^c^: One *E. coli* isolate from chicken meat harbours both *qnrB19* and *qnrS1* genes. ^d^: one *E. coli* isolate harbours both *fosA3* and *fosA7*. ^e^: one *E. coli* isolate harbours both *fosL1* and *fosA7*, another *E. coli* isolate harbour *fosL1* and *fosA3*. Bold *p*-values denote statistical significance at the *p* < 0.05 level.

**Table 2:** Frequency distribution of virulence factor genes common to *E. coli* isolated from pork and chicken meat.

Function	Gen	Pork (*n* = 85)	Chicken Meat (*n* = 87)	*p*-Value	OR
		%	%		
**Iron-related**					
Salmochelin siderophore	*iroN*	34.0	69.0	**<0.05**	**0.23**
Yersiniabactin siderophore	*fyuA/irp2*	19/19	26.4/23.0	>0.05	1.53
Aerobactin siderophore	*iutA/iucC*	33/33	89.6/89.6	**<0.05**	**0.05**
Siderophore receptor	*ireA*	2.4	17.2	**<0.05**	**0.11**
Periplasmic iron-binding protein	*sitA*	37.6	93.1	**<0.05**	**0.04**
Heme binding protein	*chuA*	11.8	42.5	**<0.05**	**0.18**
Iron and manganese transportation system	*sitABCD*	35.3	81.6	**<0.05**	**0.12**
**Adhesins**					
Adhesin/Invasin	*air*	3.5	20.7	**<0.05**	**0.14**
Curlin major subunit	*csgA*	100	98.9	>0.05	1.00
Major subunit P fimbriae	*papA*	3.5	9.2	>0.05	0.36
Outer membrane usher P fimbriae	*papC*	9.4	28.7	**<0.05**	**0.26**
Intimin-like adhesin fdec	*fdeC*	90.6	95.4	>0.05	0.46
EAEC heat-resistant agglutinin	*hra*	8.2	35.6	**<0.05**	**0.16**
Type I fimbriae	*fimH*	96.5	94.3	>0.05	1.67
Long polar fimbriae	*lpfA*	56.5	47.1	>0.05	1.45
Adherence protein	*iha*	2.4	29.9	**<0.05**	**0.06**
**Protectins**					
Outer membrane protease	*ompT*	52.9	88.5	**<0.05**	**0.15**
Increased serum survival	*iss*	55.3	95.4	**<0.05**	**0.06**
Lipoprotein Nlpl precursor	*nlpl*	100.0	98.9	>0.05	2.97
Microcin C	*cvaC*	23.5	46.0	**<0.05**	**0.36**
Outer membrane protein complement resistance	*traT*	78.8	97.7	**<0.05**	**0.06**
Anchored protein KpsE/Group II capsule biosynthesis genes	*kpsE/kpsMII*	1.2	20.7	**<0.05**	**0.05**
Putative type I secretion outer membrane protein	*etsC*	28.2	70.1	**<0.05**	**0.17**
Tellurium-resistance protein	*terC*	100.0	100.0	>0.05	1.00
Colicin IA	*cia*	20.0	26.4	>0.05	0.70
Colicin IB	*cib*	8.2	10.3	>0.05	0.78
Colicin B	*cba*	9.4	24.1	**<0.05**	**0.33**
Colicin E	*cea*	23.5	23.0	>0.05	1.03
Colicin M activity	*cma*	21.2	58.6	**<0.05**	**0.19**
Bacteriocins	*mchF*	27.1	39.1	>0.05	0.58
	*mchB/mchC*	7.1	3.4	>0.05	2.13
**Toxins**					
Avian *E. coli* haemolysin	*hlyE*	92.9	92.0	>0.05	1.15
Haemolysin F	*hlyF*	29.4	77.0	**<0.05**	**0.12**
Heat-stable enterotoxin EAST-1	*astA*	37.6	49.4	>0.05	0.62
**Others**					
Serine protease autotransporters of *Enterobacteriaceae* (SPATE)	*tsh*	21.2	50.6	**<0.05**	**0.26**
	*vat*	2.3	4.6	>0.05	0.50
	*pic*	2.3	1.1	>0.05	2.07
Invasion of brain endothelial cells	*aslA*	35.3	73.6	**<0.05**	**0.20**
Glutamate decarboxylase (tolerance to acid pH)	*gad*	100.0	100.0	>0.05	1.00
Homologs *Shigella flexneri* SHI-2 pathogenicity island gene *shiA*	*shiA*	10.6	57.5	**<0.05**	**0.09**
	*shiB*	2.3	40.2	**<0.05**	**0.04**
AraC negative regulator	*anr*	70.6	74.7	>0.05	0.81
Hemolysin expression modulator Hha (previous rmoA)	*hha*	25.9	27.6	>0.05	0.92
*Salmonella* HilA homolog	*eilA*	3.5	21.8	**<0.05**	**0.13**
Tia invasion determinant	*tia*	8.2	50.6	**<0.05**	**0.09**
Positive regulator of the conjugal transfer operon	*traJ*	67.1	63.2	>0.05	1.19

Bold *p*-values denote statistical significance at the *p* < 0.05 level.

## Data Availability

Data may be available upon reasonable request.

## Supplementary Materials

The following supporting information can be downloaded at: https://www.mdpi.com/article/10.3390/pathogens15040438/s1, Table S1: HPCIA-resistant *E. coli* isolates—WGS data.

## Abbreviations

The following abbreviations are used in this manuscript:

AMR	Antimicrobial resistance
APEC	Avian pathogenic *E. coli*
CIP	Ciprofloxacin
CTX	Cefotaxime
EC-C	*E. coli* isolated from chicken meat
EC-P	*E. coli* isolated from pork
ESBL	Extended-spectrum β-lactamase
ExPEC	Extraintestinal pathogenic *E. coli*
FOS	Fosfomycin
IPEC	Intestinal pathogenic *E. coli*
HPCIA	High-priority and critically important antimicrobial agents
HiRC	High-Risk Clones
PMQR	Plasmid-mediated quinolone resistance
QRDR	Quinolone resistance-determining regions
UPEC	Uropathogenic *E. coli*
WHO	World Health Organization
3GC	Third-generation cephalosporins
